# Angiotensin(1–7) attenuates visceral adipose tissue expansion and lipogenesis by suppression of endoplasmic reticulum stress via Mas receptor

**DOI:** 10.1186/s12986-022-00716-x

**Published:** 2022-12-16

**Authors:** Chifa Ma, Tingting Shi, Lini Song, Jingyi Liu, Mingxia Yuan

**Affiliations:** 1 Department of Endocrinology, Beijing Friendship Hospital, Capital Medical University, Beijing, 100050 China; 2 Department of Endocrinology, Beijing Tongren Hospital, Capital Medical University, Beijing, 100730 China

**Keywords:** ACE2, Ang(1–7), Mas, Visceral adipose tissue expansion, Lipogenesis, Endoplasmic reticulum stress

## Abstract

**Background:**

White adipose tissue can be classified based on its location as subcutaneous and visceral fat, and the latter accumulation is reported to be more detrimental to metabolism. Endoplasmic reticulum (ER) stress has been demonstrated to regulate lipogenesis. The peptide angiotensin(1–7) [Ang(1–7)], which can be produced from angiotensin II (AngII) by angiotensin-converting enzyme 2 (ACE2), plays its role through Mas receptor, also participates in the regulation of lipid metabolism in adipose tissue, however, whether ER stress is involved in the mechanism remains unclear. Therefore, we aimed to explore the role of Ang(1–7) pathway in regulating visceral adipose tissue expansion and ER stress.

**Methods:**

*ACE2* knockout (KO), *Mas* KO and C57BL/6 J mice were fed with high fat diet. Db/db mice were treated with either normal saline, Ang(1–7) or Ang(1–7) combined with Mas receptor inhibitor A779 using mini osmotic pumps. Fat mass was weighted, fat distribution was evaluated by MRI, and lipid profile and adipokines in epididymal adipose tissue were measured by ELISA kits, and histology of epididymal adipose tissue was also analyzed in multiple animal models. Additionally, differentiated 3T3-L1 cells were pre-loaded with palmitic acid to induce ER stress, then treated with drugs as those administrated to db/db mice. ER stress and lipogenesis related proteins in mice adipose and differentiated 3T3L-1 cells were analyzed by Western blot.

**Results:**

*ACE2* or *Mas* KO mice exhibited increased visceral adipose tissue, adipocyte size and protein expression of lipogenesis and ER stress related markers in epididymal adipose tissue compared to wild-type mice. Db/db mice treated with Ang(1–7) displayed decreased visceral fat mass, adipocyte size and protein expression of lipogenesis and ER stress markers in epididymal adipose tissue compared to those treated with normal saline, while A779 partly attenuated these effects. Additionally, Ang(1–7) improved ER stress and lipogenesis markers in differentiated 3T3-L1 cells pre-loaded with palmitic acid.

**Conclusions:**

Our findings indicated that Ang(1–7) attenuated visceral adipose tissue expansion and lipogenesis by suppression of ER stress via Mas receptor. The present study provides a potential perspective for Ang(1–7) for the therapeutics of obesity and related disorders.

**Supplementary Information:**

The online version contains supplementary material available at 10.1186/s12986-022-00716-x

## Introduction

Obesity is one of the most common metabolic disorders worldwide, and it’s closely associated with diabetes, hypertension, cardiovascular diseases and a number of cancers, making it a worldwide health problem and challenge. Obesity is characterized by excessive white adipose tissue accumulation. White adipose tissue can be classified based on its location as subcutaneous and visceral fat, and the latter accumulation is reported to be more detrimental to metabolism [1, 2], thus inhibiting excessive visceral adipose tissue expansion would contribute to prevent obesity and associated comorbidities.

Lipogenesis is the process by which fatty acids derived from lipoproteins are esterified with glycerol to synthetize triglycerides (TG) which are then stored in lipid droplets [3]. The endoplasmic reticulum (ER) is the organelle responsible for regulating calcium homeostasis, lipid metabolism and protein synthesis. ER plays an important role in sensing cellular stress and mediating Unfolded Protein Response (UPR) [4], which could adjust the protein-folding capacity of the cell to sustain the cell's secretory function and mitigate ER stress by the activating transcription factor 6, inositol requiring enzyme-1, and PKR-like endoplasmic reticulum kinase signaling pathways. ER is also the major site for the synthesis of sterols and phospholipids, and many enzymes and regulatory proteins involved in lipid metabolism reside in the ER [5]. ER plays vital roles in regulation of lipogenesis and lipid droplets formation, and ER stress has emerged as a vital regulator of lipid biosynthesis and adipokine secretion [6]. Moreover, activation of ER stress is closely associated with many metabolic diseases such as obesity and non-alcoholic fatty liver diseases [7, 8], thus ER stress has been a highly promising therapeutic target for these diseases.

The renin angiotensin system (RAS) plays important roles in regulating blood pressure, fluid and electrolyte homeostasis [9]. In recent years, it has also been found that RAS plays a vital role in obesity and insulin resistance [10, 11]. The peptide angiotensin(1–7) [Ang(1–7)], which can be produced from angiotensin II (AngII) by angiotensin-converting enzyme 2 (ACE2), plays its role through Mas receptor and thereby exerts increasing nitric oxide production and inhibitory effects on inflammation [12–14]. All of the main RAS components including ACE2 and Mas receptor are present in adipose tissue [15]. It was reported that mice with total *Mas* ablation demonstrated dyslipidemia and a significant increase in abdominal fat mass [16], while Ang(1–7) treated rats demonstrated decreased total fat mass along with decreased serum TG [17], however, whether ER stress was involved in the underling mechanism remained unclear. Our previous studies showed that ACE2 plays an important role in intramuscular fat regulation and hepatic steatosis partly via ER stress improvement [18, 19]. And, Zhang et al. showed that ACE2/Ang(1–7)/Mas axis may ameliorate ER stress in lung and heart [20, 21], suggesting that ACE2/Ang(1–7)/Mas axis participates in the regulation of ER stress. However, no studies have focused on the effects of this axis on ER stress in white adipose tissue. In the present study, we aimed to explore the role of Ang(1–7) pathway in regulating of visceral adipose tissue expansion and ER stress.

## Materials and methods

### Animals

All animals were treated in accordance with the protocol approved by the Ethics Committee of Animal Research at Beijing Tongren Hospital, Capital Medical University, Beijing, China. Animals were housed in controlled-temperature rooms (22 °C) with a 12:12-h dark–light cycle.

ACE2 cleaves Ang II to produce Ang(1–7), which acts mainly through the Mas receptor. Thus, the deletion of *ACE2* or *Mas* would affect the expression levels or the action of Ang(1–7), and *ACE2* knockout (KO) and *Mas* KO mice were chosen to explore the role of the lack of Ang(1–7) on metabolism. Male *ACE2* KO mice(8 weeks of age) and their age and sex-matched C57BL/6 J wild-type (WT) littermates, and male *Mas* KO mice and their age and sex-matched C57BL/6 J WT littermates were obtained from the Nanjing Biological Medicine Research Institute (*n* = 5–7/group), and were fed with high-fat diet (HFD)(60 kcal% fat) (Research Diets, New Brunswick, NJ, USA) for 8 weeks, and body weight was measured every week.

Male db/db mice at 8 weeks of age were purchased from the Nanjing Biological Medicine Research Institute, and were fed with HFD(60 kcal% fat) (Research Diets, New Brunswick, NJ, USA) (*n* = 5/group). Db/db mice were concomitantly treated with either normal saline (NS), Ang(1–7) (576 µg/kg/day; H-1715, Bachem, USA), or Ang(1–7) combined with Mas receptor inhibitor A779 (1152 µg/kg/day; H-2888, Bachem, USA) for 4 weeks using mini osmotic pumps (Alzet-Durect, Cupertino, CA, USA Model ^#^1004) placed subcutaneously [22], and body weight was measured every week.

### Fat analysis by magnetic resonance imaging

Body fat distribution of *ACE2* KO, *Mas* KO and WT mice was assayed by magnetic resonance imaging (MRI) (Varian 7 T/160 mm animal MRI scanner) equipped with a gradient coil system producing a gradient of up to 400 mT/m in each of the three dimensions. The calculation of fat volume is based on the fat segmentation method [23].

### Tissue collection

All mice were sacrificed after anesthesia. Subcutaneous and visceral fat tissues were rapidly removed and weighed, immediately frozen in liquid nitrogen, and stored at − 80 °C for posterior analysis.

### Determination of lipid profile, adipokines and Ang(1–7) in epididymal adipose tissue

Frozen epididymal adipose tissue from different mice models was allowed to thaw to room temperature and homogenized by macerating with a disposable glass pipet. Adipose total cholesterol (TC) and TG were measured by enzymatic colorimetric method using commercial kits (Beijing Labo Biotech, CO, LTD). Adipose leptin and adiponectin were measured by ELISA kits (eBioscience, USA). In addition, Ang(1–7) levels in epididymal adipose tissue of WT and *ACE2* KO mice were also detected by ELISA kits (Cloud-Clone Corp., China).

### Histology

Epididymal adipose tissue from different mice models was fixed in buffered formalin, embedded in paraffin, sectioned, mounted on slides, deparaffinized and stained with hematoxylin/eosin to evaluate the adipocyte morphology according to a standard protocol [24]. Images were captured on a slide scanner (3DHISTECH, Pannoramic MIDI), and the representative visions were chosen by the Pannoramic viewer imaging system. For adipocyte size measurements, two images per mice were analyzed using the Pannoramic viewer imaging system.

### Cell culture and treatments

3T3-L1 pre-adipocytes were purchased from Cell Resource Center, IBMS, CAMS/PUMC, China. Cells were maintained in Dulbecco’s Modified Eagle’s Medium (DMEM, GIBCO, Carlsbad, CA, USA) supplemented with 10% calf serum and penicillin–streptomycin (100 U/ml, GIBCO) at 37 °C with 5% CO_2_ under humidified conditions. Upon confluence, the growth medium was changed the following day and replaced with differentiation medium consisting of DMEM, 0.5 mM 3-isobutly-1-methylxanthine (IBMX, cat. No. 2547, Sigma Aldrich, St. Louis, MO, USA), 1 uM dexamethasone (cat. No. D1756, Sigma Aldrich), 10% fetal bovine serum (FBS, GIBCO), and 10ug/ml insulin (I5500-100 mg, Sigma Aldrich) for 2 days. After additional 2 days in DMEM containing 10% FBS and 10 μg/ml insulin, the growth medium was replaced with DMEM supplemented with 10% FBS for another 2–4 days. Oil Red O staining was performed to confirm the differentiation of adipocytes.

The differentiated cells were treated with NS as control or pre-loaded with 400 µM of palmitic acid (PA, Sigma-Aldrich, St. Louis, MA, USA) for 24 h to induce ER stress [25], then treated with NS, 10^–9^ mmol/L Ang(1–7) or both Ang(1–7) and 10^–6^ mmol/L A779 for 24 h [26]. Oil Red O staining was performed to assess lipid accumulation.

### Oil Red O staining

Differentiated 3T3-L1 cells were washed three times with PBS and then fixed with 4.0% formaldehyde for 25 min. Subsequently, cells were stained with freshly diluted Oil Red O solution for 20 min at room temperature. Finally, cells were washed with double distilled water, and visualized by light microscopy and photographed.

### Western blot

Epididymal adipose tissue and 3T3-L1 cells were homogenized in RIPA buffer, and protein concentration in lysates was assessed by the BCA method using a commercially available kit (Beyotime, China). Equal amounts of protein were resolved by 10% SDS-PAGE gel, transferred electrically onto PVDF membranes (Millipore, Billerica, MA, USA). After blocking with 5% nonfat milk, primary antibodies were added overnight at 4 °C. The antibodies targeted the following proteins: β-Actin (4970, CST, USA), ACE2 (Sc-20998, Santa Cruz, USA), Mas (AAR-013, Alomone labs, Israel), Fatty Acid Synthase (FAS) (3180, CST), acetyl-CoA carboxylase α (ACCα) (Sc-30212, Santa Cruz), sterol regulatory element-binding protein-1c (SREBP-1c) (Sc-366, Santa Cruz), C/EBP homologous protein (CHOP) (2895, CST), Glucose regulated protein 78 (GRP78) (ab21685, Abcam, England), activating transcription factor 4 (ATF4) (11,815, CST). Then the membranes were incubated with horseradish peroxidase-conjugated secondary antibodies at room temperature for 1.5 h. The bands were finally detected with ECL Detection Regents (Bio-Rad, USA).

### Statistical analysis

Data were expressed by mean ± SEM. Different groups were compared by Student t test or one-way ANOVA (with Bonferroni post-hoc tests to compare replicate means). Prism 5 (GraphPad Software, San Diego, CA) was used for all statistical analyses. *P* < 0.05 (two-side) indicated a statistically significant difference.

## Results

### *ACE2* deletion tended to increase visceral fat and led to increased adipose leptin in mice

**Fig. 1:**
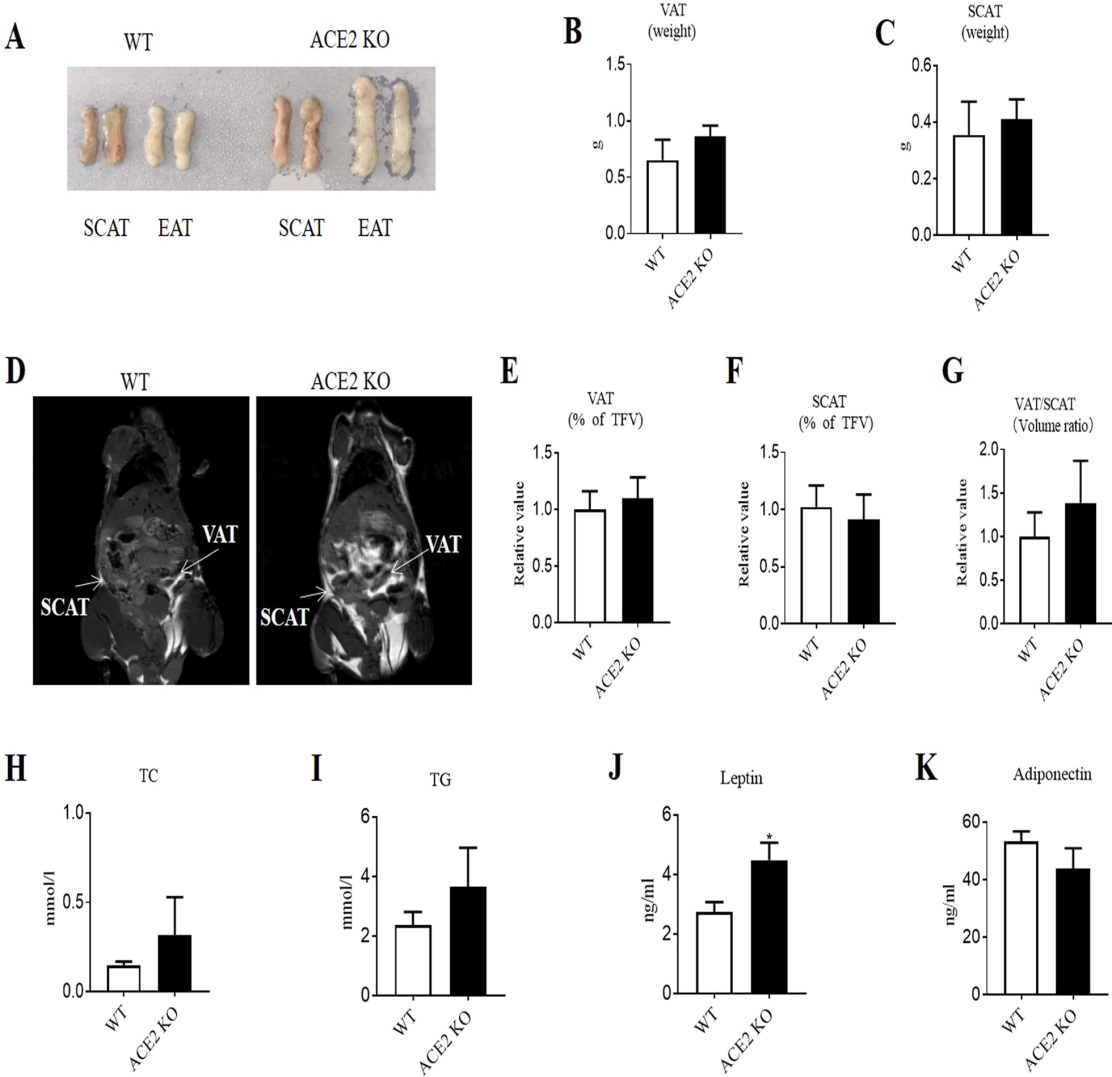
*ACE2* deletion tended to increase visceral fat and led to increased adipose leptin in mice. **A** Image depicting SCAT and EAT from WT and *ACE2* KO mice. **B** Weight of VAT. **C** Weight of SCAT. **D** MRI analysis of VAT and SCAT. Representative images for VAT and SCAT. **E** Relative value of the proportion of VAT volume. **F** Relative value of the proportion of SCAT volume. **G** Relative value of the VSR. **H** Levels of TC in EAT. **I** Levels of TG in EAT. **J** Levels of leptin in EAT. **K** Levels of adiponectin in EAT. Data are represented as mean ± SME from *n* = 3–5 each group; *p < 0.05 versus WT group. WT, wild-type; ACE2, angiotensin-converting enzyme 2; KO, knock out; SCAT, subcutaneous adipose tissue; EAT, epididymal adipose tissue; MRI, magnetic resonance imaging; TFV, total white fat volume; VAT, visceral adipose tissue; VSR, visceral to subcutaneous fat ratio; TC, total cholesterol; TG, triglycerides

Because the peptide Ang(1–7) can be produced from Ang II by ACE2 enzyme, the ACE2 is an upstream regulating enzyme of Ang(1–7), and *ACE2* KO mice exhibited decreased plasma Ang(1–7) [27]. We used the *ACE2* KO mice to explore the effects of deficiency of *ACE2* on visceral adipose tissue expansion and ER stress. We analyzed ACE2 expression in epididymal adipose tissue by Western blot, which indicated significant reduction of ACE2 protein expression in *ACE2* KO mice (Additional file 1: Fig. S1A). As expected, *ACE2* KO mice exhibited decreased Ang(1–7) levels in epididymal adipose tissue (Additional file 1: Fig. S1B). The *ACE2* KO mice showed similar body weight gain to that of WT mice after fed with HFD for 8 weeks (Additional file 1: Fig. S2A). However, histological analysis of white adipose tissue showed that *ACE2* KO mice had a substantial increase in epididymal fat mass (Fig. 1A). The weight of visceral fat tended to increase in *ACE2* KO mice compared to that of WT mice (Fig. 1B), and the difference of the weight of subcutaneous fat was not notable (Fig. 1C). The proportion of visceral fat volume estimated by MRI showed the same trend as visceral fat weight (Fig. 1D and E), and the difference of proportion of subcutaneous fat volume was not notable (Fig. 1F). The results of MRI analysis also showed that the visceral to subcutaneous fat volume ratio tended to increase in *ACE2* KO mice compared with WT mice (Fig. 1G). The levels of TC, TG and adiponectin in epididymal adipose tissue showed no significant differences (Fig. 1H, I and K), while the levels of leptin were significantly increased in epididymal adipose tissue of *ACE2* KO mice (Fig. 1J).

### ACE2 deletion induced adipocyte hypertrophy, increased lipogenesis and ER stress in epididymal adipose tissue in mice

To further explore the mechanism of visceral adipose tissue expansion, epididymal adipose tissue was used to explore the effects of *ACE2* deletion on adipocyte size, lipogenesis and ER stress.

**Fig. 2:**
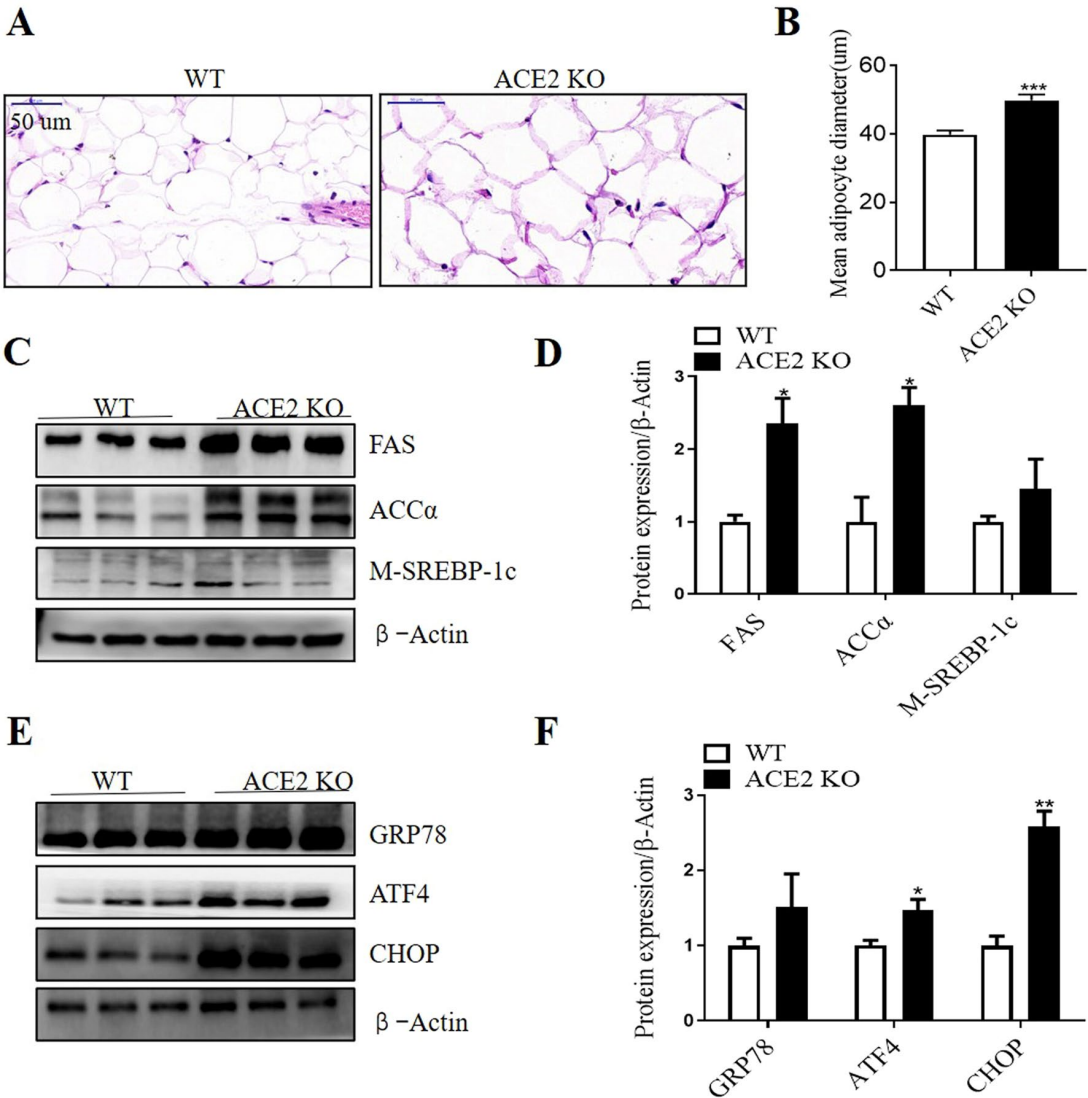
*ACE2* deletion induced adipocyte hypertrophy, increased lipogenesis and ER stress related markers in epididymal adipose tissue in mice. **A** Hematoxylin/eosin staining for epididymal adipose tissue from WT and *ACE2* KO mice. Bars indicate a length of 50um. **B** The mean diameter of epididymal adipocytes. **C** and **D** Representative Western blot and relative protein levels of lipogenesis related markers in epididymal adipose tissue. **E** and **F** Representative Western blot and relative protein levels of ER stress related markers in epididymal adipose tissue. Data are represented as mean ± SEM from *n* = 3. *p < 0.05, **p < 0.01, ***P < 0.001vs WT group. WT, wild-type; ACE2, angiotensin-converting enzyme 2; KO, knock out; FAS, fatty acid synthase; ACCα, acetyl-CoA carboxylase α; M-SREBP-1c, mature sterol regulatory element-binding protein-1c; GRP78, glucose regulated protein 78; ATF4, activating transcription factor 4; CHOP, C/EBP homologous protein

The H&E staining and quantification of fat cell diameter indicated that the epididymal adipocyte size of the *ACE2* KO mice increased substantially compared with that from WT mice (Fig. 2A and B). The expression of lipogenesis related proteins was examined by Western blot. Compared with WT mice, the protein levels of FAS and ACCα were significantly increased in the epididymal fat tissue of *ACE2* KO mice (Fig. 2C and D).

To further explore the underlying mechanism of *ACE2* deletion on lipogenesis, we examined whether *ACE2* deletion regulated ER stress in epididymal adipose tissue. The protein levels of several ER stress related markers in epididymal adipose tissue were examined by Western blot. As shown in Fig. 2E and F, the protein expression of ATF4 and CHOP was significantly upregulated in *ACE2* KO mice compared with WT mice.

### Mas deletion led to increased visceral adipose tissue mass and adipose leptin in mice

**Fig. 3:**
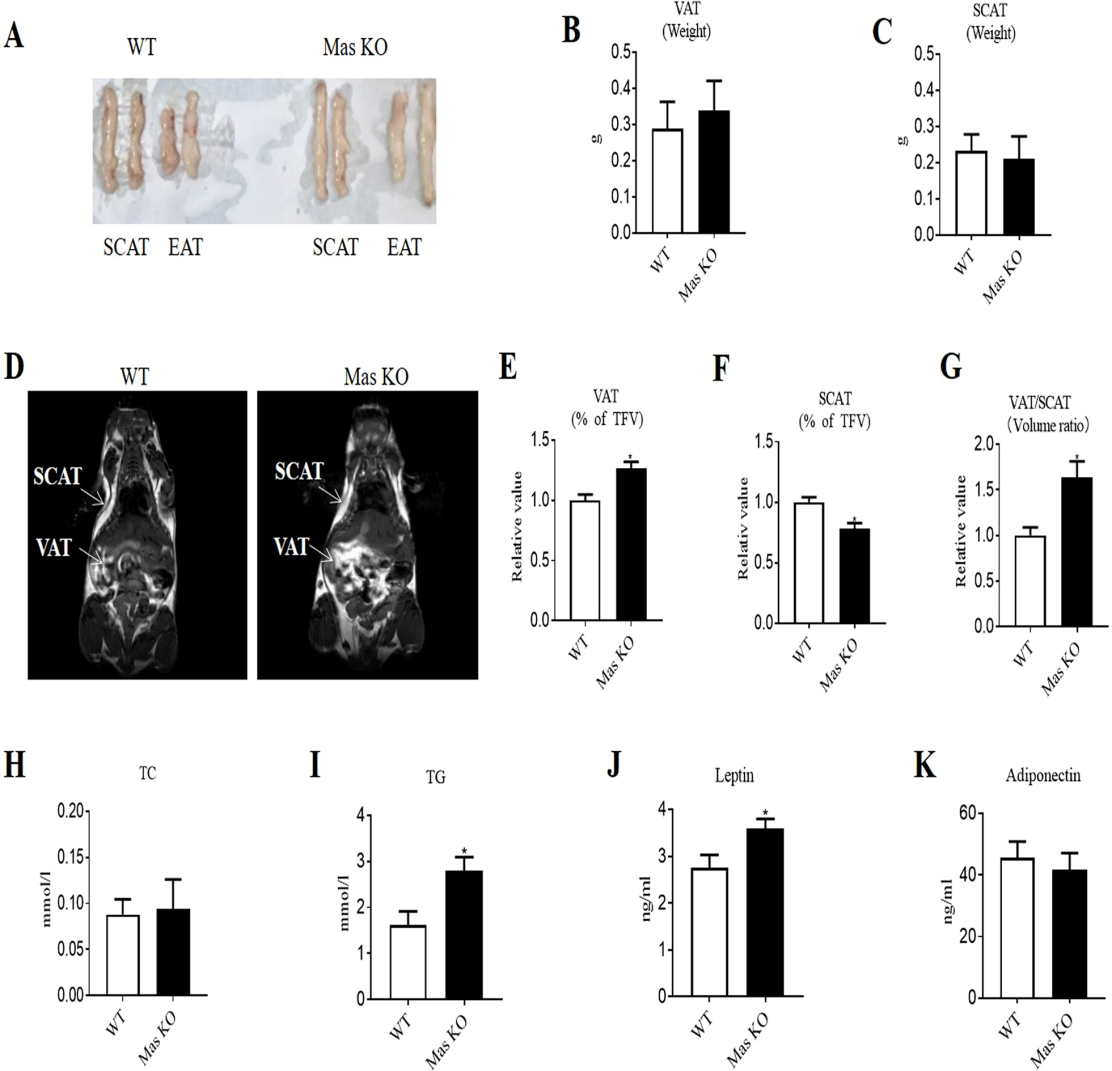
*Mas* deletion led to increased visceral adipose tissue mass and adipose leptin in mice. **A** Image depicting SCAT and EAT from WT and *Mas* KO mice. **B** Weight of VAT. **C** Weight of SCAT. **D** MRI analysis of VAT and SCAT. Representative images for VAT and SCAT. **E** Relative value of the proportion of VAT volume. **F** Relative value of the proportion of SCAT volume. **G** Relative value of VSR. **H** Levels of TC in EAT. **I** Levels of TG in EAT. **J** Levels of leptin in EAT. **K** Levels of adiponectin in EAT. Data represents mean ± SD from *n* = 3–5. *p < 0.05 versus WT group. WT, wild-type; KO, knock out; SCAT, subcutaneous adipose tissue; EAT, epididymal adipose tissue; MRI, magnetic resonance imaging; TFV, total white fat volume; VAT, visceral adipose tissue; VSR, visceral to subcutaneous fat ratio; TC, total cholesterol; TG, triglycerides

We also used the *Mas* KO mice to explore the effects of the deficiency of the receptor of Ang(1–7) on visceral adipose tissue expansion and ER stress*.* We analyzed Mas protein expression in adipose tissue by Western blot, which indicated significant reduction of Mas protein expression in *Mas* KO mice and confirmed *Mas* deletion (Additional file 1: Fig. S1C). *Mas* KO mice tended to gain more body weight compared to that of WT mice after fed with HFD for 8 weeks, but the difference did not reach significance (Additional file 1: Fig. S2B). We also examined the adipose pads in WT and *Mas* KO mice, and found that epididymal fat mass was greater in *Mas* KO than WT mice (Fig. 3A). The weight of visceral fat tended to increase in *M*as KO mice (Fig. 3B), while the weight of subcutaneous fat tended to decrease (Fig. 3C). MRI scan analysis showed that the proportion of visceral fat volume and visceral to subcutaneous fat volume ratio was significantly increased (Fig. 3D, E and G), and the proportion of subcutaneous fat volume was significantly decreased in *Mas* KO mice compared with WT mice (Fig. 3F).

Then the levels of TC, TG, adiponectin and leptin in epididymal adipose tissue were analyzed. The levels of TG and leptin were significantly increased in epididymal adipose tissue of *Mas* KO mice compared to those in WT mice (Fig. 3I and J), while the difference of the levels of TC and adiponectin did not reach significance (Fig. 3H and K).

### Mas deletion induced adipocyte hypertrophy, increased lipogenesis and ER stress in epididymal adipose tissue in mice

**Fig. 4:**
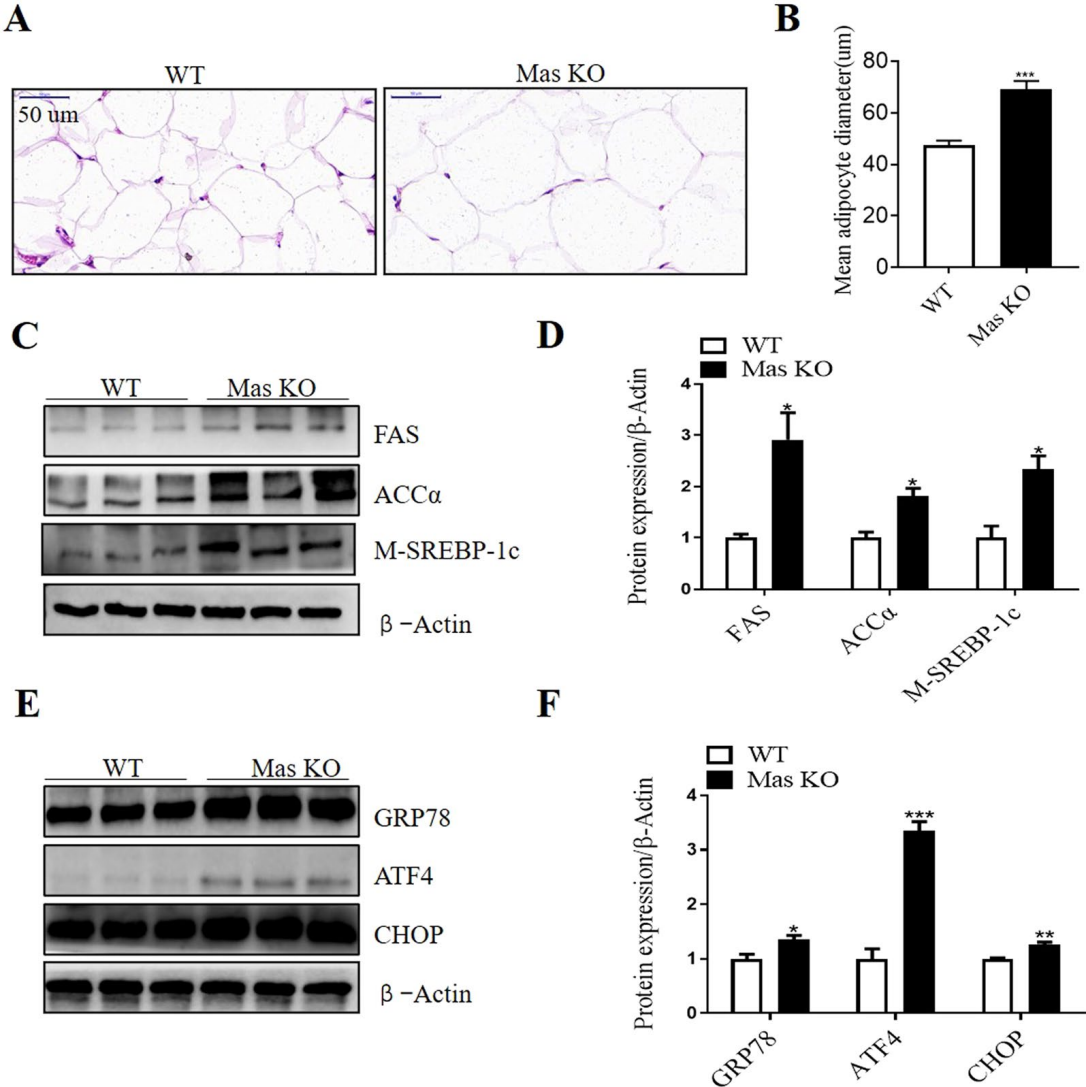
*Mas* deletion induced adipocyte hypertrophy, increased lipogenesis and ER stress related markers in epididymal adipose tissue in mice. **A** Hematoxylin/eosin staining for epididymal adipose tissue from WT and *Mas* KO mice. Bars indicate a length of 50um. **B** The mean diameter of epididymal adipocytes. **C** and **D** Representative Western blot and relative protein levels of lipogenesis related markers in epididymal adipose tissue. **E** and **F** Representative Western blot and relative protein levels of ER stress related markers in epididymal adipose tissue. Data are represented as mean ± SEM from n = 3. *p < 0.05, **p < 0.01, ***p < 0.001 versus WT group. WT, wild-type; KO, knock out; FAS, fatty acid synthase; ACCα, acetyl-CoA carboxylase α; M-SREBP-1c, mature sterol regulatory element-binding protein-1c; GRP78, glucose regulated protein 78; ATF4, activating transcription factor 4; CHOP, C/EBP homologous protein

The H&E staining and quantification of adipocyte diameter indicated that the epididymal adipocyte size of *Mas* KO mice increased substantially compared with that from WT mice (Fig. 4A and B). Then the protein expression of lipogenesis related markers was examined by Western blot in epididymal fat tissue. Compared with WT mice, the protein expression of M-SREBP-1c, FAS and ACCα was significantly increased in the epididymal fat tissue from *Mas* KO mice compared with WT mice (Fig. 4C and D).

Next, we examined whether *Mas* deletion regulated ER stress in the epididymal adipose tissue. Protein expression of ER stress related markers in the epididymal adipose tissue was examined by Western blot. The protein levels of GRP78, ATF4 and CHOP were significantly increased in *Mas* KO mice compared with WT mice (Fig. 4E and F).

### Ang(1–7) treatment decreased visceral adipose tissue mass and adipose leptin in db/db mice

**Fig. 5:**
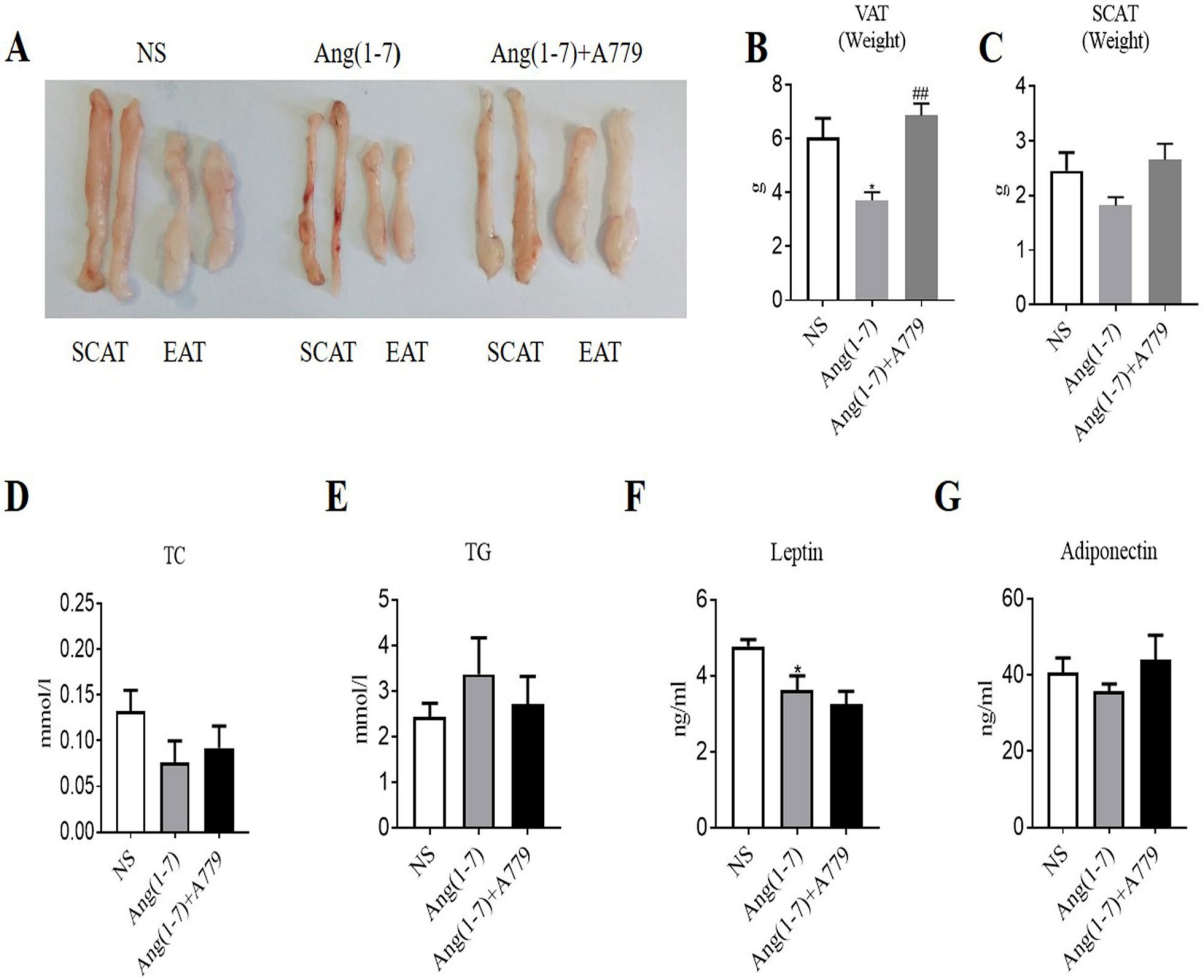
Ang(1–7) treatment decreased visceral adipose tissue mass and adipose leptin in db/db mice. **A** Image depicting SCAT and EAT from db/db mice treated with NS, Ang(1–7), and Ang(1–7) combined with A779. **B** Weight of VAT. **C** Weight of SCAT **D** Levels of TC in EAT. **E** Levels of TG in EAT. **F** Levels of leptin in EAT. **G** Levels of adiponectin in EAT. Data are represented as mean ± SEM from n = 3–5. *p < 0.05 versus NS group, ^##^*P* < 0.01 versus Ang(1–7) group by one-way ANOVA. Ang(1–7), angiotensin(1–7); NS, normal saline; SCAT, subcutaneous adipose tissue; EAT, epididymal adipose tissue; VAT, visceral adipose tissue; TC, total cholesterol; TG, triglycerides

To explore the effects of Ang(1-7) on visceral adipose tissue expansion and ER stress, db/db mice were assigned into three treatment groups [NS, Ang(1–7), and Ang(1–7) combined with A779]. The mice in Ang(1–7) group tended to gain less body weight compared to that in NS and Ang(1–7) combined with A779 groups, but the difference was not significant.(Additional file 1: Fig. S2C). Anatomical analysis of the epididymal and subcutaneous adipose tissue showed that Ang(1–7) treatment decreased epididymal fat mass compared with NS treatment, while A779 inhibited these effects (Fig. 5A). The visceral adipose weight in Ang(1–7) group was significantly decreased compared with NS group, while the weight in Ang(1–7) combined with A779 group was significantly increased compared with Ang(1–7) group(Fig. 5B), and weight of subcutaneous adipose tissue had the similar tendency (Fig. 5C). In addition, Ang(1–7) treatment decreased the level of leptin in epididymal adipose tissue (Fig. 5F), while levels of TC, TG and adiponectin in adipose did not differ among the groups (Fig. 5D, E and G).

### Ang(1–7) treatment induced decreased adipocytes size, lipogenesis and ER stress in epididymal adipose tissue in db/db mice

**Fig. 6:**
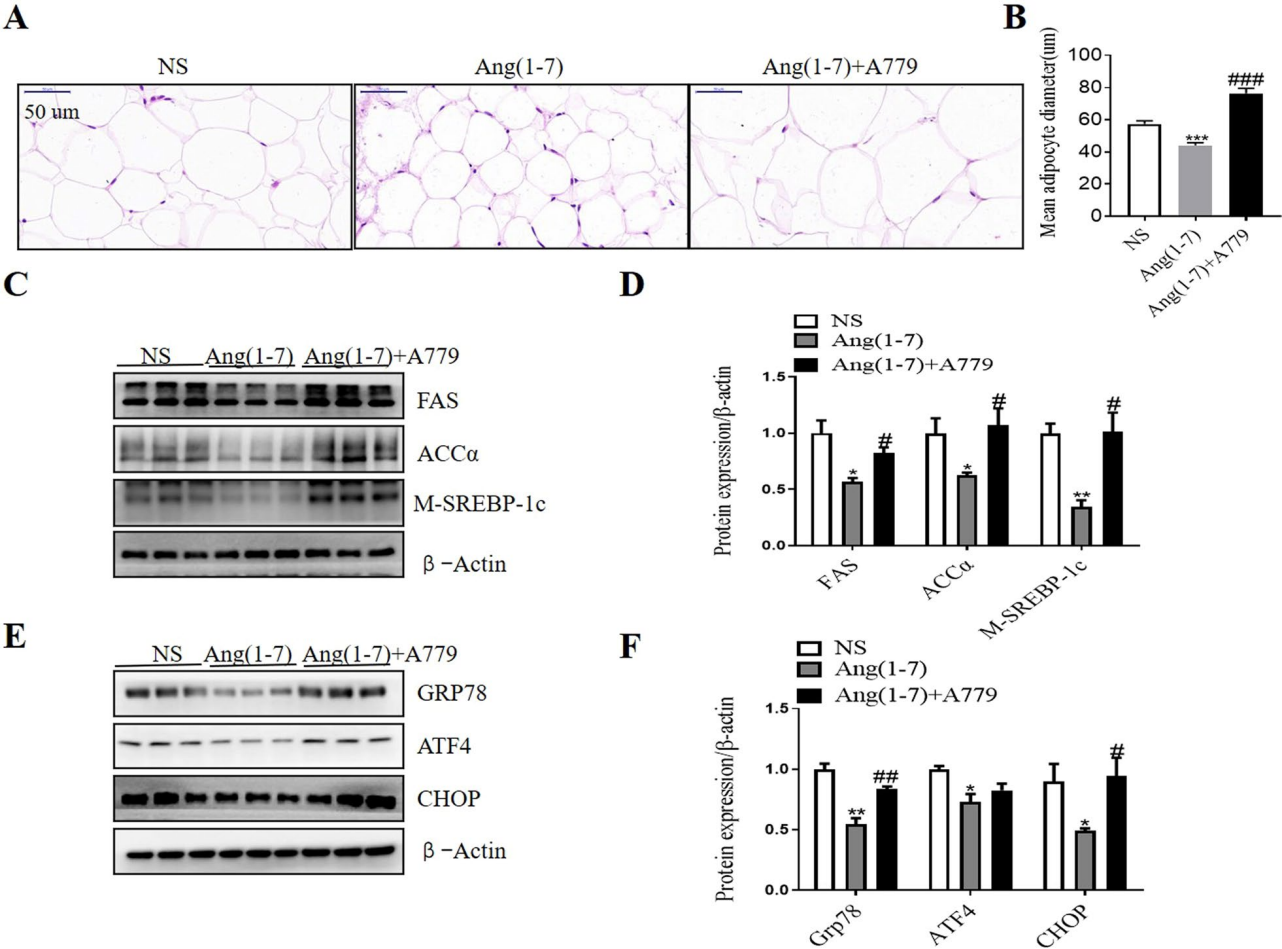
Ang(1–7) treatment induced decreased adipocytes size, lipogenesis and ER stress related markers in epididymal adipose tissue of db/db mice. **A** Hematoxylin/eosin staining for epididymal adipose tissue from db/db mice treated with NS, Ang(1–7), and Ang(1–7) combined with A779. Bars indicate a length of 50um. **B** The mean diameter of epididymal adipocytes. **C** and **D** Representative Western blot and relative protein levels of lipogenesis related markers in epididymal adipose tissue. **E** and **F** Representative Western blot and relative protein levels of ER stress related markers in epididymal adipose tissue. Data are represented as mean ± SEM from n = 3. *p < 0.05, **p < 0.01, ***p < 0.001 versus NS group; ^#^*p* < 0.05, ^##^*p* < 0.01, ^###^*p* < 0.001 versus Ang(1–7) group by one-way ANOVA. Ang(1–7), angiotensin(1–7); NS, normal saline; FAS, fatty acid synthase; ACCα, acetyl-CoA carboxylase α; M-SREBP-1c, mature sterol regulatory element-binding protein-1c; GRP78, glucose regulated protein 78; ATF4, activating transcription factor 4;CHOP, C/EBP homologous protein

H&E staining indicated that the adipocyte size in epididymal fat tissue of Ang(1–7) treated db/db mice was markedly smaller than that of NS group, while the adipocyte size in Ang(1–7) combined with A779 group was significantly bigger than that in Ang(1–7) group (Fig. 6A and B).

Then we examined the expression of m-SREBP-1c, FAS and ACCα by Western blot in the epididymal fat tissue from mice. Compared with NS group, the protein expression of m-SREBP-1c, FAS and ACCα was significantly decreased in the epididymal adipose tissue of Ang(1–7) treated db/db mice, while A779 treatment inhibited these effects (Fig. 6C and D).

Next, we examined whether Ang(1–7) treatment regulated ER stress in the epididymal adipose tissue. Protein levels of ER stress related markers including GRP78, ATF4 and CHOP in the epididymal adipose tissue were examined by Western blot. The protein expression of GRP78, ATF4 and CHOP was significantly decreased in Ang(1–7) treated db/db mice compared with NS treated mice, while A779 treatment partly attenuated these effects (Fig. 6E and F).

### Ang(1–7) treatment induced decreased lipogenesis and ER stress in differentiated 3T3-L1 cells

**Fig. 7:**
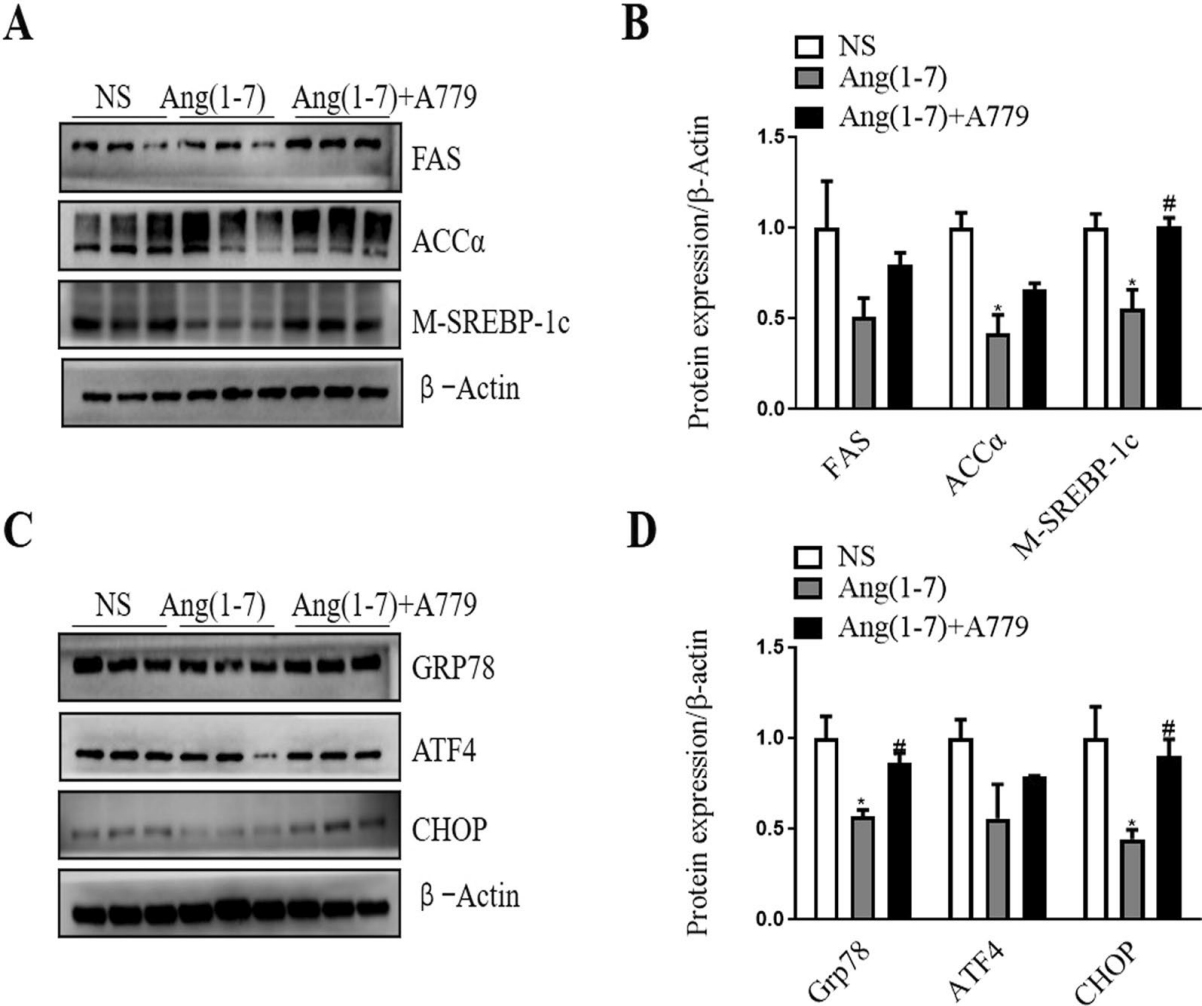
Ang(1–7) treatment induced decreased lipogenesis and ER stress related markers in differentiated 3T3-L1 cells pre-loaded with palmitic acid. The differentiated cells were pre-loaded with 400 µM of palmitic acid for 24 h to induce ER stress, then treated with NS, 10^–9^ mmol/L Ang(1–7) or both Ang(1–7) and 10^–6^ mmol/L A779 for 24 h. **A** and **B** Representative Western blot and relative protein levels of lipogenesis related markers. **C** and **D** Representative Western blot and relative protein levels of ER stress related markers. Data are represented as mean ± SEM. *p < 0.05 versus NS group; ^#^*p* < 0.05 versus Ang(1–7) group by one-way ANOVA. Ang(1–7), angiotensin(1–7); NS, normal saline; FAS, fatty acid synthase; ACCα, acetyl-CoA carboxylase α; M-SREBP-1c, mature sterol regulatory element-binding protein-1c; GRP78, glucose regulated protein 78; ATF4, activating transcription factor 4; CHOP, C/EBP homologous protein

To confirm whether Ang(1–7) treatment could directly regulate ER stress, differentiated 3T3-L1 cells were treated with NS or pre-loaded with an ER stress inducer of PA, then treated with NS, Ang(1–7) or Ang(1–7) together with A779. Oil Red O staining indicated that PA treatment induced increased cell size and lipid accumulation (Additional file 1: Fig.S3).In cells pre-loaded with PA, Oil Red O staining showed that Ang(1–7) treatment decreased cell size and lipid accumulation compared to NS treatment, while A779 treatment partly attenuated the effects (Additional file 1: Fig.S3). Western blot further confirmed that Ang(1–7) treatment significantly decreased the protein expression of lipogenesis (ACCα and M-SREBP1c) (Fig. 7A and B) and ER stress markers (GRP78 and CHOP) (Fig. 7C and D) compared to NS treatment, and A779 treatment partly attenuated the effects. These results were consistent with data in db/db mice.

## Discussion

ACE2 is an upstream regulating enzyme of Ang(1–7), and Mas is the receptor of Ang(1–7), thus the deletion of *ACE2* or *Mas* gene may actually inhibit the action of Ang(1–7). In the present study, we found that *ACE2* KO or *Mas* KO mice exhibited increased visceral adipose tissue, higher leptin levels, larger adipocyte size, and upregulated lipogenesis and ER stress related proteins in epididymal adipose tissue compared to that of WT mice fed with HFD. However, the *ACE2* KO mice showed similar body weight gain to that of WT mice after fed with HFD for 8 weeks, which may be explained by the important role ACE2 playing in the expression of neutral amino acid transporters in the gut [28]. Moreover, db/db mice treated with Ang(1–7) exhibited decreased visceral adipose tissue, lower leptin levels, smaller adipocyte size, and downregulated lipogenesis and ER stress related proteins in epididymal adipose tissue compared to those treated with NS, while those treated with A779 simultaneously could partly antagonized above protective effects. Additionally, Ang(1–7) improved ER stress markers and attenuated lipogenesis in differentiated 3T3-L1 cells pre-loaded with ER stress inducer of PA, and A779 partly attenuated these effects. Our results suggested that Ang(1–7) pathway could attenuated ER stress and visceral adipose tissue expansion of different mice models for the first time.

According to the functions, color, mitochondrial content, location, vascularization and structure, adipose tissue can be divided into three types, namely white, brown and beige adipose tissue. The major function of the former is to store extra energy in the form of TG, while the primary function of the latter two is to dissipate energy in the form of heat [29]. Our previous study indicated that the ACE2/Ang(1–7) pathway regulated brown fat function and systemic energy metabolisms [27], we further confirmed this pathway’s role in white adipose tissue in our present study, enriching its role in obesity and related disease. White adipose tissue can be classified as visceral and subcutaneous fat according to the location. Visceral fat accumulation is reported to be more detrimental to metabolism than subcutaneous fat [1, 2], because the former is closely associated with risk factors for cardiovascular disease [30]. We found that *ACE2* KO or *Mas* KO mice exhibited increased visceral fat tissue compared to WT mice fed with HFD, while db/db mice treated with Ang(1–7) exhibited decreased visceral fat tissue compared to those treated with NS. It was reported that *Mas* KO mice also demonstrated an increase in abdominal fat mass [16], while chronic Ang(1–7) treatment could decrease total fat mass in mice [17], and ACE2 activator DIZE decreased epididymal and retroperitoneal adipose tissue weights [31], and these studies further supported our findings. It is well-known that increased adipocyte number (hyperplasia) or size (hypertrophy) leads to fat accumulation and obesity. However, hyperplasia is decreased during extreme obesity, potentially due to reduced adipogenesis [32]. Therefore, inhibiting adipocyte hypertrophy would play an important role in preventing adipose tissue expansion in obesity. We found that *ACE2* KO and *Mas* KO mice exhibited larger adipocyte size in epididymal adipose compared to WT mice fed with HFD, while Ang(1–7) treated db/db mice exhibited smaller adipocyte size compared to those treated with NS, suggesting that the mass of epididymal adipose depended on the change of adipocyte size to some extent in these ACE2/Ang(1–7)/Mas axis altered mice models. Lipogenesis in adipocytes is the process to synthetize TG which are then stored in lipid droplets, which would make the adipocyte size larger. SREBP-1c, ACCα and FAS were important lipogenesis related markers, and we found that *ACE2* and *Mas* KO could upregulate the protein expression of these markers compared to WT mice in epididymal adipose tissue, while Ang(1–7) could downregulate them compared to those treated with NS, and the Mas antagonist A779 had opposite effects, which were consistent with previous studies reporting the similar findings in liver and muscle [18, 19]. We also founded that Ang(1–7) could downregulate lipogenesis and attenuate lipid accumulation in differentiated 3T3-L1 cells in our present study, and A779 inhibited above effects. Moreover, it was reported that activation of ACE2 by oral DIZE treatment decreased lipogenesis related gene transcription as well as epididymal adipose tissue weight [31]. Collectively, Ang(1–7) could attenuate lipogenesis via Mas receptor and consequently may lead to smaller adipocyte size and decreased epididymal adipose mass.

More and more studies suggested that obesity is correlated with ER stress in adipose tissue. ER stress has been evidenced to participate in the modulation of lipogenesis in adipose tissue [6]. A study reported that activation of ER stress increased SREBP-1c in human mature adipocytes [33], and activation of SREBPs could consequently trigger lipogenesis related markers such as ACCα and FAS [6]. However, when ER stress was inhibited, lipogenesis related markers were significantly downregulated [33]. Our previous study found that ACE2 preserve skeletal muscle lipid metabolism and non-alcoholic fatty liver diseases partly via regulation of ER stress [18, 19], however, the potential role of ACE2/Ang(1–7)/Mas axis on adipose lipid metabolism whether involved in regulation of ER stress remained uncertain. Our present study found that *ACE2* or *Mas* KO mice exhibited increased expression of ER stress related proteins in epididymal fat compared to WT mice fed with HFD, while db/db mice treated with Ang(1–7) exhibited decreased ER stress related markers in epididymal adipose tissue compared to those treated with NS, and the Mas antagonist A779 had opposite effects. To exclude the influence of  obesity on ER stress [34] and confirm the direct effects of Ang(1–7) pathway, we also performed vitro experiment and further found that Ang(1–7) could improve ER stress markers in the differentiated 3T3-L1 cells pre-loaded with the ER stress inducer of PA, demonstrating the change of ER stress at least partly by Ang(1–7) pathway. Collectively, we found that Ang(1–7) pathway participated in ER stress suppression by in vitro and in vivo studies. Moreover, stimulation of ER stress was reported to active lipogenesis [6, 33], and we found that Ang(1–7) attenuated ER stress and lipogenesis, thus Ang(1–7) may attenuate visceral fat expansion by suppression of ER stress. In addition, the weight and volume of visceral fat, and visceral to subcutaneous fat ratio was changed in different mice models, which confirmed the effects of ACE2/Ang(1–7)/Mas axis on fat distribution, however, the detail mechanism needs to be further explored.

Adipokine secretion is also regulated by ER stress in adipose tissue [35, 36]. Leptin, one of the most abundant adipokines, was reported to be regulated by ER stress and Ang(1–7) pathway. One the one hand, ER stress inhibitor 4-phenylbutyric acid (4-PBA) could potentially ameliorate leptin signaling in db/db mice [37], while different ER stress inducers markedly inhibited leptin-induced STAT3 phosphorylation [38]. On the other hand, it was reported that *Mas* deficiency in FVB/N mice presented increased leptin [16]. Our results suggested that *ACE2* or *Mas* deletion induced increased levels of adipose leptin and ER stress, while Ang(1–7) had opposite effects. Considering the role of Ang(1–7) pathway on ER stress and leptin secretion, we hypothesized that Ang(1–7) pathway may reduce leptin secretion by suppression of ER stress. Although, Fig. 5F showed that adipose leptin in Ang(1–7) combined with A779 treatment group was also decreased compared to that in Ang(1–7) treatment group, not entirely supporting this hypothesis. However, this phenomenon may be explained by the fact that multiple factors contribute to regulation of leptin secretion [39]. Thus, further studies focused on leptin secretion are needed. Adiponectin, another adipokine, is also regulated by ER stress. Specifically, it was reported that increased ER stress reduced adiponectin levels in 3T3-L1 cells, and when ER stress was suppressed, adiponectin level was restored [35]. Santos SH et al. reported increased circulating Ang(1–7) upregulated adiponectin production in transgenic rats [10]. Moreover, we found that Ang(1–7) could ameliorate ER stress, thus Ang(1–7) may regulate adiponectin by ER stress. However, we did not see significant differences in adipose adiponectin between or among different mice models, although there existed significant differences in ER stress markers, and further studies are needed to explore the mechanism of adiponectin secretion.

We demonstrated that Ang(1–7) pathway could attenuated visceral adipose tissue expansion by different mice models for the first time, and the mechanism may involve the downregulation of lipogenesis induced by suppression of ER stress in visceral adipose tissue, which further provided a potential strategy for treating abdominal obesity. Some limitations of the current study should also be noted. Firstly, *ACE2* or *Mas* deletion in mice was not adipocyte specific, which could not rule out the possibility effects from systemic action, therefore, conditional KO mice models are needed to further confirm the study. Secondly, we did not record the food intake for all in vivo models in our present study, which was important for obesity assessment, however, our previous study found ACE2/Ang(1–7)/Mas pathway had no significant effects on food intake [27].

## Conclusion

It was demonstrated that Ang(1–7) could attenuated visceral adipose tissue expansion and adipocyte hypertrophy via Mas receptor, and the mechanism may due to the downregulation of lipogenesis caused by suppression of ER stress. The present study provides a potential perspective for Ang(1–7) for the therapeutics of obesity and related disorders.

## Supplementary Information

**Additional file 1.** Supplementary figures.

## Data Availability

The data that support the findings of this study are available from the corresponding author on reasonable request.

## Abbreviations

ER: Endoplasmic reticulum
RAS: Renin angiotensin system
Ang(1–7): Angiotensin(1–7)
ACE2: Angiotensin-converting enzyme 2
KO: Knock out
HFD: High-fat diet
WT: Wild-type
HFD: High-fat diet
NS: Normal saline
MRI: Magnetic resonance imaging
TC: Total cholesterol
TG: Triglyceride
DMEM: Dulbecco’s modified eagle’s medium
IBMX: 3-Isobutly-1-methylxanthine
FBS: Fetal bovine serum
FAS: Fatty acid synthase
ACCα: Acetyl-CoA carboxylase α
M-SREBP-1c: Mature sterol regulatory element-binding protein-1c
CHOP: C/EBP homologous protein
GRP78: Glucose regulated protein 78
ATF4: Activating transcription factor 4
