# Supplementary material for: Angiotensin(1–7) attenuates visceral adipose tissue expansion and lipogenesis by suppression of endoplasmic reticulum stress via Mas receptor
Source: Nutr Metab (Lond). 2022 Dec 16;19:82. doi: 10.1186/s12986-022-00716-x (PMC9758942; doi:10.1186/s12986-022-00716-x)
Supplement: Supplementary file 1 — Additional file 1. Supplementary figures. [file 12986_2022_716_MOESM1_ESM.docx]

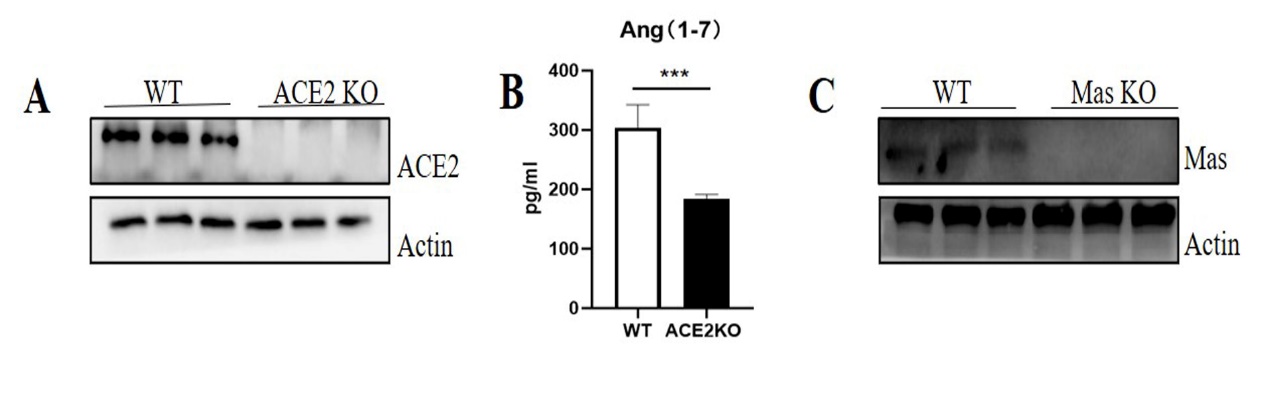


**Fig. S1** Quantification of ACE2, Ang(1-7) and Mas in epididymal adipose tissue. (A) Western blot image of ACE2 in epididymal adipose tissue from WT and *ACE2* KO mice (n=3). (B) Epididymal adipose tissue levels of Ang(1-7) in WT and *ACE2* KO mice, as determined by ELISA (n=4). (C) Western blot image of Mas in epididymal adipose tissue from WT and *Mas* KO mice (n=3). ^***^P< 0.001vs WT group. WT, wild-type; ACE2, angiotensin-converting enzyme 2; KO, knock out; Ang(1-7), angiotensin(1-7).

.


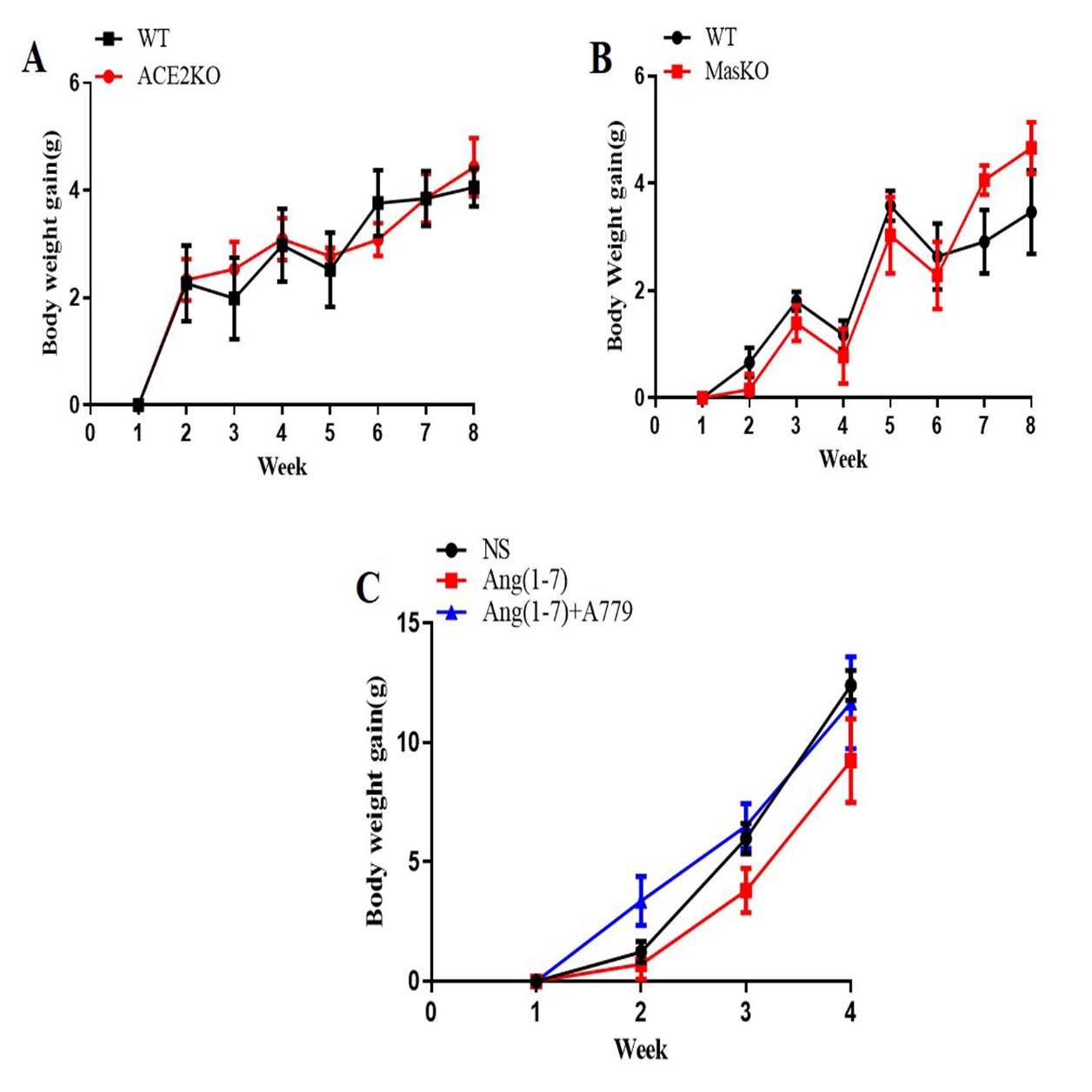


**Fig. S2** Body weight gain of different mice models. (A) Body weight gain of WT and *ACE2* KO mice after fed with high-fat diet for 8 weeks (n=5-7). (B) Body weight gain of WT and *Mas* KO mice after fed with high-fat diet for 8 weeks (n=5-6). (C) Body weight gain of db/db mice after treated with NS, Ang (1-7), and Ang(1-7) combined with A779 for 4 weeks(n=4). Data are represented as mean± SEM. WT, wild-type; ACE2, angiotensin-converting enzyme 2; KO, knock out; Ang(1-7), angiotensin(1-7); NS, normal saline.


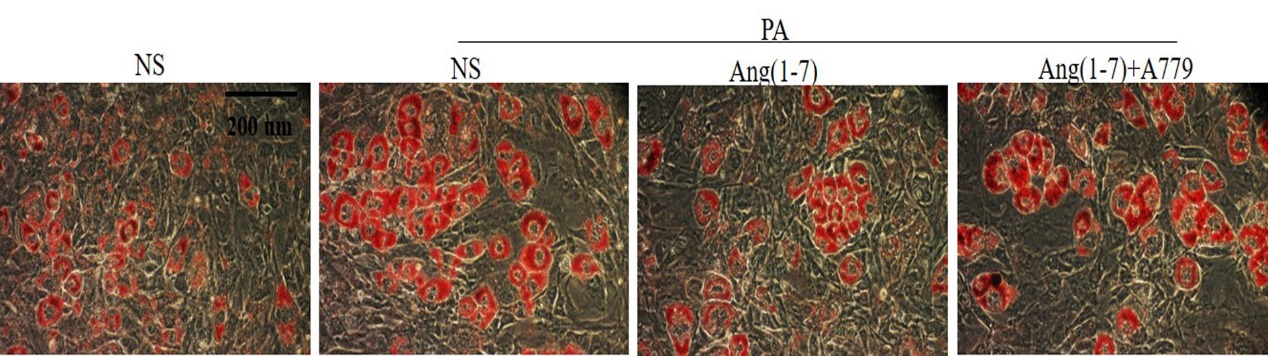
 **Fig. S3** Effect of PA and Ang(1-7) on lipid accumulation in differentiated 3T3-L1 cells, as detected by Oil red O staining. The representative pictures of Oil Red O staining. Bars indicate a length of 200 µm. The differentiated cells were treated with NS as control or pre-loaded with 400 µM of PA for 24h to induce ER stress, then treated with NS, 10^-9^mmol/L Ang(1-7) or both Ang(1-7) and 10^-6^ mmol/L A779 for 24h. Ang(1-7), angiotensin(1-7); NS, normal saline; PA, palmitic acid.
